# Practice of COVID-19 prevention measures and its factors in long-distance truck drivers of Tigray

**DOI:** 10.11604/pamj.2024.48.96.40378

**Published:** 2024-07-11

**Authors:** Aregawi Gebreyesus, Asqual Gebreslase

**Affiliations:** 1 Department of Epidemiology, College of Health Science, Mekelle University, Mekelle, Tigray, Ethiopia

**Keywords:** COVID-19, prevention, practice, Tigray

## Abstract

**Introduction:**

long-distance truck drivers are a population group that moves in and out of a country and can meet with different individuals and can easily expose to COVID-19. Therefore, this study aimed to assess the level of practice and associated factors towards COVID-19 prevention measures in long-distance truck drivers of Tigray.

**Methods:**

this is a cross-sectional study conducted on 350 long-distance truck drivers recruited by systematic random sampling in the Mekelle entry point from July 5^th^ to July 20^th^ 2020. Variables with a p-value ≤0.30 in the bivariate regression analysis were entered into the final model of logistic regression to recognize factors. All associations with the practice of COVID-19 prevention were tested for statistical significance with alpha set at the 0.05 level.

**Results:**

around 293 (83.7%) with a 95% CI of (79.7-87.6%) of the long-distance truck drivers who participated in this study had good practice towards COVID-19 prevention measures. Having a previous test for COVID-19 is positively significantly associated with good practice towards COVID-19 prevention methods. However, individuals who had a history of COVID-19-like symptoms in the last four months and who have non-communicable diseases are 78% and 84% of the time less likely to practice COVID-19 prevention measures respectively.

**Conclusion:**

based on this study, the practice of COVID-19 prevention measures among the long-distance truck drivers of Tigray was very good. The results of this study suggest that more emphasis should be placed on drivers who have non-communicable diseases and providing COVID-19-like symptoms to the drivers.

## Introduction

Severe acute respiratory syndrome coronavirus 2 (SARS-CoV-2) is a new coronavirus that emerged in Wuhan, China in 2019. It has caused an exceptional worldwide pandemic of respiratory illness, labeled ‘coronavirus diseases 2019’ (COVID-19) [1]. Several studies show that individual factors such as age, gender, or occupation influence travel behaviors and movement patterns. In addition to this, long-distance truck drivers around the world have been identified as a high-risk group for this pandemic and are consequently the targets for prevention and education-based intervention [1-3]. Multiple nations have declared total blockage of their country and limited public movement. However, for long truck drivers become difficult to apply that due need for societal economic supplies. Long-distance truck drivers are a population moves in and out of the country and can meet with different individuals in different countries. Due to this reason, truck drivers are rendered vulnerable to disease due to their iterant lifestyle [4]. Then, to minimize the spread of the virus among drivers and become source of infection for the remained population, World Health Organization (WHO) recommend preventive measures such as: proper hand washing and utilizing sanitizer, vehicle disinfection, utilizing face masking, and physical distancing [5]. These preventive measures are very effective if applied appropriately.

However, studies show that even though there is good knowledge among the participants, practice towards COVID-19 preventive measures is poor in most relative to knowledge and attitude. This also differs among different groups of individuals [6,7]. In contradiction of this, a survey done among 6910 participants of high-risk group in China shows more than 90% of them were having good knowledge, practice, and positive attitude [8]. When we came to Africa; a study done in Nigeria among 284 high-risk groups shows that 69.7% of them were not practicing the preventive measures for COVID-19 [9]. A study done in Ethiopia, among the high-risk group (healthcare workers), also shows that of 397 healthcare professionals, only 63.5% of them were having good practices toward COVID-19 prevention measures [10].

Supporting this, a study done in Uganda shows that a total of 89,224 have been tested and from 442 positive individuals the majority 317 (71.8%) were long-distance truck drivers [11]. Even though long-distance truck drivers are among the most vulnerable individuals to contract the coronavirus, to the best of our research review these parts of the population are the most forgotten, and no more studies are conducted to show their practice level towards COVID-19 to design appropriate measures. Thus, this study aimed to assess the level of practice and associated factors towards COVID-19 prevention measures in long-distance truck drivers of Tigray.

## Methods

**Study design and setting:** this study was conducted using a community-based cross-sectional study design from July 5 to July 25, 2020, in Mekelle City, the capital city of Tigray. Mekelle city had two (2) land crossing entry points (from Djibouti and other neighboring regions) and one international airport entry. Especially the Djibouti-Mekelle entry point is the main source of business for the whole Tigray that enters from other countries and sea ports. Regarding COVID-19, the city had 28 quarantine centers and two (2) isolation and treatment centers.

**Study population:** our study population was the long-distance truck drivers who were entering from Djibouti to Tigray through the Mekelle entry point. All consenting long truck distance drivers participated by filling out the data collection forms and volunteering for venous blood according to the prepared standard operating procedure. All individuals who didn´t give informed consent were excluded from the study.

**Sample size and procedure:** the sample size was determined for both objectives using Epi-Info software. Using previous studies done in different countries, we determined the sample size for both objectives. We took the sample size of the first objective and determined to use a single proportion formula due to the higher number. In a study conducted in the study in Allam *et al*. 2020 the proportion of practice toward COVID-19 prevention measures was 47% (p) from [12], and this prevalence was taken in our sample size estimation with a 5% margin of error (d), 95% level of confidence, Z α/2= 1.96, and 10% no response. Based on these assumptions the minimum sample size required is 389.

**Data collection tool and technique:** information on socio-demographic status, clinical information, and other epidemiologic data was collected by adopting the WHO tool and previous studies in a way that matches the study requirements [9,12-17]. Data were collected using a structured and pre-tested questionnaire by trained interviewers using an electronic data collection form. During the data collection period, supervisors were recruited to monitor the completeness of data and to ensure other quality-related issues.

**Variables and measurements:** practice towards prevention measures for COVID-19 was an outcome variable, and it was measured using four practice-level questions, four used a Likert scale (three categories): yes/always (score 1), yes/sometimes (0), and no (0). The outcome variable was measured using the following: proper hand washing and utilizing sanitizer, vehicle disinfection, utilizing face masking, and physical distancing. Then the questionnaire was also pre-tested beforehand the real data collection. Lastly, by saying above/equal and below the mean the response was classified into “good practice (coded as 1)” and “poor practice (coded as 0)” respectively. The data collectors visited and checked the practice using direct observation.

The reliability of the practice questionnaires was checked by computing Cronbach´s α, which was found to be high (> 0.7), which was 0.795, and then the questionnaire was also pre-tested before the real data collection [17]. The independent variables include socio-demographic factors (age, sex, educational status, occupation, contact history); a history of illness and symptoms; and underlying situations.

**Data processing and analysis:** after the data was coded and entered using the double data entry by two data clerks, the reliability of the entered data was double-checked by relating the two independently and cleaned using Epi-Data version 3.1. Then, the data was exported to the SPSS statistical software version 22.0 for analysis. Descriptive statistical analysis such as measures of central tendency, simple frequencies, and measures of variability was used to describe the characteristics. Then the information was presented using frequencies, summary measures, and tables. For assessing practice for prevention measures of COVID-19. Four Likert scale questions were used. Each question has the alternative “yes/always” (score as 1), “yes/sometimes" (score as 0), and “no” (score as 0). Later, the responses were dichotomized into “good practice,” if the respondents reported “yes/always” and “poor practice,” if the respondents reported either “yes/sometimes” or “no” with practice component questions.

Those variables with a p-value ≤0.30 in the bivariate regression analysis were entered into the final model to recognize factors associated with the practice of prevention measures of COVID-19 and statistical significance was declared at a 95% CI and p-value < 0.05. Collinearity between variables was weighed by looking at the values of variance inflation factors (VIFs). VIF > 10 is assumed to be indicative of the presence of multi-collinearity. Model fitness was also determined at a p-value > 0.05 using the Hosmer-Lemeshow test.

**Ethical consideration:** ethical approval was obtained from the Scientific Ethical Review Office (SERO) of the Ethiopian Public Health Institute. Letters of permission were also written by the Tigray Bureau of Health. Written consent was obtained from study participants/long-distance truck drivers and informed about the right to refuse to answer part of the questionnaire or the entire questionnaire. Confidentiality was maintained by using the questionnaire anonymously.

## Results

**Level of practice and characteristics of respondents:** a total of 350 study participants were enrolled in this study and made a response rate of 90%. The mean ± SD age of the respondents was 37.4 ± 9.04 with a range of 19 up to 60 years old. Around 293 (83.7%) with a 95% CI of (79.7-87.6%) of them have good practices towards COVID-19 prevention measures. No significant difference was shown between age categories and ethnicity in the practice of COVID-19 prevention measures. A significant difference is shown between educational level and practice of COVID-19 prevention measures (χ^2^(1)= 0.039, P< 0.05). Around 193 (87.3%) or the majority of the drivers who complete secondary school having good practice (Table 1).

**Table 1:** socio-demographic characteristics and practice of COVID-19 prevention in long-distance truck drivers, June-July 2020 (n=350)

Variable	Classification	Practice		X^2^	DF (P-value)
		Good N (%)	Poor N (%)		
Age (yrs.)	19-30	98(89.1)	12(10.9)	4.9	3(0.18)
	31-40	97(80.2)	24(19.8)		
	41-50	68(85)	12(15)		
	51-60	30(76.9)	9(23.1)		
Origin	Tigrayan	149(82.3)	32(17.7)	0.53	1(0.47)
	Non-Tigrayan	144(85.2)	25(14.8)		
Education	Primary	84(76.4)	26(23.6)	6.48	2(0.039)
	Secondary	193(87.3)	28(12.7)		
	College+	16(84.2)	3(15.8)		

DF: degrees of freedom

Drivers who have a contact history and who don't have a contact history have not shown a significant difference in COVID-19 prevention methods. The same for drivers who took a test for COVID-19 or not. But significant difference was seen between drivers who experienced and didn´t experience COVID-19-like symptoms in the previous 4 months (χ^2^(1)=21.4, P<0.001) towards COVID-19 prevention methods. Drivers who have and do not have noncommunicable diseases (NCDs) also tend to show significant discrepancies towards COVID-19 prevention methods at (χ^2^(1)=16.11, P<0.001) (Table 2).

**Table 2:** medical and personal characteristics and practice of COVID-19 prevention in long-distance truck drivers, June-July 2020 (n=350)

Variable	Classification	Practice		X^2^	DF (P-value)
		Good N (%)	Poor N (%)		
Contact history	Yes	2 (75)	1 (25)	0.39	1 (1.0)
	No	291 (83.6)	57 (16.3)		
Tested for COVID-19	Yes	237 (86.5)	37(13.5)	7.16	1 (0.07)
	No	56 (73.7)	20 (26.3)		
*COVID-19 symptoms in the last 4 months	Yes	19 (55.9)	15 (44.1)	21.4	1 (0.000)
	No	274 (86.7)	42 (13.3)		
∫Having NCDs	Yes	5(41.7)	7 (58.3)	16.11	1 (0.000)
	No	288 (85.2)	50 (14.8)		
Having HIV/tuberculosis	Yes	0	0	Na	Na
	No	293 (83.7)	57 (16.3)		

* Fever, chills, fatigue, muscle ache, sore throat, cough, runny nose, shortness of breath, wheezing, chest pain, nausea, loss of smell/taste; ∫hypertension, diabetic mellitus, heart disease, chronic respiratory diseases; DF: degrees of freedom

**Factors associated with the practice of COVID-19 prevention measures:** all variables that were found statistically significant with the outcome variable in chi-square (X^2^) at the p-value of ≤0.25 were used in multivariate regression analysis. After those statistically significant bivariate regression/tabulations entered into the multivariate regression analysis, significant associated factors were declared at the p-value of ≤0.05.

Having a previous test for COVID-19 is significantly associated with good practice towards COVID-19 prevention methods at (AOR 3.08, 95% CI 1.55-6.08; P<0.05). But drivers who had a history of COVID-19-like symptoms in the last four months and who have NCDs are 78% and 84% of the time less likely to practice COVID-19 prevention measures with (AOR 0.22, 95% CI 0.1-0.49; P<0.05), (AOR 0.16 95% CI 0.04-0.58; P<0.05) respectively (Table 3).

**Table 3:** factors associated with the practice of COVID-19 prevention in long-distance truck drivers, June-July 2020 (n=350)

Variable	Classification	Practice		COR (95% CI)	AOR (95% CI)
		Good, N (%)	Poor, N (%)		
Age	19-30	98(89.1)	12(10.9)	2.45(0.94-6.37)	2.61 (0.91-7.48)
	31-40	97(80.2)	24(19.8)	1.2 (0.51-2.9)	1.24 (0.48-3.24)
	41-50	68(85)	12(15)	1.7 (0.65-4.46)	1.70 (0.59-4.86)
	51-60	30(76.9)	9(23.1)	1	1
Education	Primary	84(76.4)	26(23.6)	0.61 (0.17-2.24)	0.59 (0.14-2.57)
	Secondary	193(87.3)	28(12.7)	1.29 (0.35-4.72)	1.27 (0.31-5.13)
	College and above	16(84.2)	3(15.8)	1	1
Origin	Tigrayan	149(82.3)	32(17.7)	0.81 (0.46-1.43)	0.65 (0.35-1.24)
	Non-Tigrayan	144(85.2)	25(14.8)	1	1
Tested for COVID-19	Yes	237 (86.5)	37(13.5)	0.44 (0.24-0.81) *	3.08 (1.55-6.08) *
	No	56 (73.7)	20 (26.3)	1	1
Hx of COVID-19 like symptoms	Yes	19 (55.9)	15 (44.1)	5.15 (2.43-10.91) *	0.22 (0.1-0.49) *
	No	274 (86.7)	42 (13.3)	1	1
Having NCDs	Yes	5(41.7)	7 (58.3)	8.06 (2.46-26.41) *	0.16 (0.04-0.58) *
	No	288 (85.2)	50 (14.8)	1	1

* Statistically significance of p-value <0.05; COR: crude odds ratios; AOR: adjusted odds ratio; NCDs: non-communicable diseases

## Discussion

This study showed that the level of practice towards COVID-19 prevention among long-distance truck drivers of Tigray was 83.7% with a 95% CI of (79.7-87.6%). Regarding the factors; a history of tests for COVID-19, having a history of COVID-19-like symptoms, and having NCDs were found to be significantly associated with the outcome variable of this study.

Based on this study, the level of practice towards COVID-19 prevention measures among long-distance truck drivers is 83.7%. This means the majority of drivers (83.7%) of long-distance trucks drivers have a good practice towards COVID-19 prevention measures regularly. This is consistent with studies done in Saudi Arabia and China [6,8], and higher than from a study done in Ethiopia and Nigeria [9,10]. This might be due to the long truck drivers getting better awareness in multiple COVID-19 screening stations daily inside and outside the country.

Regarding the factors associated with the practice, those who previously tested for COVID-19 were 3 times more likely to practice COVID-19 prevention measures than those who didn't have a test. This might be because those who took a test might be well aware of the prevention measures and their health. Because at a testing time clients are always given a health education related to COVID-19. On the other hand, long-distance truck drivers who had a history of COVID-19-like symptoms in the last four months were 78% less likely to practice COVID-19 prevention measures compared to those who didn't experience COVID-19-like symptoms. This might be due to a misunderstanding of the nature of the virus and if the symptom wasn´t much difficult, they can think the virus will not cause them any trouble. The other reason might be respondents who had COVID-19-like symptoms think and take the symptoms as COVID-19 symptoms, and after relieving the symptoms they might feel freedom because they may think they are secure and immune from COVID-19. Lastly, long-distance truck drivers who had NCDs tend to practice 84% fewer preventive measures towards COVID-19 than those who had no NCDs. This may need other and further studies to look at its consistency. But the reason might be because of a lack of awareness about the disease and its co-morbidities.

**Limitations of the study:** since the study is a cross-sectional study temporal relationship cannot be established. In addition to this, some data used in this study were self-reported, which might suffer from reporting bias or interviewer bias. As the outcome variable is measured using four practice questions and later converted to a binary variable (good/poor practice), it might have an under/over-estimation of the final result and the findings of the factors.

## Conclusion

Based on this study, the practice of COVID-19 prevention measures among the long-distance truck drivers of Tigray is better. The results of this study suggest that more emphasis should be placed on drivers who have NCDs and providing COVID-19-like symptoms to the drivers.

### What is known about this topic

- *Practice towards COVID-19 prevention measures in long-distance drivers was 83.7%;*
- *Those having a previous test for COVID-19 are positively significantly associated with good practice towards COVID-19 prevention methods;*
- *Individuals who had a history of COVID-19-like symptoms in the last four months and drivers with non-communicable diseases had poor practice*.

### What this study adds

- *This is a study done on a special exposed part of society unlike other studies;*
- *Unlike other findings, this study showed that those with non-communicable diseases had poor practice;*
- *Having a history of COVID-19-like symptoms was associated with poor practice, unlike other studies*.
